# The *γ*-secretase complex: from structure to function

**DOI:** 10.3389/fncel.2014.00427

**Published:** 2014-12-11

**Authors:** Xian Zhang, Yanfang Li, Huaxi Xu, Yun-wu Zhang

**Affiliations:** ^1^Fujian Provincial Key Laboratory of Neurodegenerative Disease and Aging Research, Institute of Neuroscience, College of Medicine, Xiamen UniversityXiamen, FJ, China; ^2^Degenerative Disease Research Program, Sanford-Burnham Medical Research InstituteLa Jolla, CA, USA

**Keywords:** *γ*-secretase, Alzheimer’s disease, anterior pharynx defective 1, nicastrin, presenilin, presenilin enhancer 2

## Abstract

One of the most critical pathological features of Alzheimer’s disease (AD) is the accumulation of β-amyloid (Aβ) peptides that form extracellular senile plaques in the brain. Aβ is derived from β-amyloid precursor protein (APP) through sequential cleavage by β- and γ-secretases. γ-secretase is a high molecular weight complex minimally composed of four components: presenilins (PS), nicastrin, anterior pharynx defective 1 (APH-1), and presenilin enhancer 2 (PEN-2). In addition to APP, γ-secretase also cleaves many other type I transmembrane (TM) protein substrates. As a crucial enzyme for Aβ production, γ-secretase is an appealing therapeutic target for AD. Here, we summarize current knowledge on the structure and function of γ-secretase, as well as recent progress in developing γ-secretase targeting drugs for AD treatment.

## Background

Alzheimer’s disease (AD) is the most prevalent aging associated neurodegenerative disorder, afflicting approximately 10% of the population over age 65 and 30–50% of the population over age 85. A subset (<10%) of AD manifests as familial early-onset AD (FAD; onset in the fourth to sixth decade) and is inherited as an autosomal dominant disorder. Mutations in the genes encoding β-amyloid precursor protein (APP) and presenilins (PS1 and PS2) are causative in the majority of FAD kindred (Goate et al., 1991; Levy-Lahad et al., 1995a; Sherrington et al., 1995; Haass and De Strooper, 1999). Because the clinicopathological features of FAD are apparently indistinguishable from sporadic AD cases, great efforts have been devoted to studying these FAD linked genes and significant progress has been made to reveal mechanisms underlying AD pathogenesis.

The presence of extracellular senile plaques in the brain is a key pathological feature of AD. Senile plaques are largely comprised of variously sized Aβ peptides, where most peptides are represented by Aβ40 and the more deleterious Aβ42 species (Glenner and Wong, 1984; Masters et al., 1985; McColl et al., 2012). Aβ is produced through sequential proteolytic processing of APP by β- and γ-secretases (Haass and Selkoe, 1993; Chami and Checler, 2012). Alternatively, cell surface APP can be cleaved by α-secretase within the Aβ peptide domain to release the non-amyloidogenic soluble APPα, which has been shown to be neuroprotective (Greenfield et al., 2000). Experimental evidence from humans, animal models, and cultured cells all suggest that Aβ is the prime culprit for AD pathogenesis: excessive Aβ triggers a cascade of neurodegenerative events resulting in the formation of neuritic plaques and intra-neuronal fibrillary tangles and neuronal loss in AD (Selkoe, 1998; Greenfield et al., 2000; Golde, 2005). However, some studies suggest that the ratio of Aβ42 to Aβ40, rather than the total amount of Aβ, exhibits better correlation with the age of FAD onset (Kumar-Singh et al., 2006). Due to the importance of Aβ generation in AD pathogenesis, γ-secretase has become an important focus in AD research and has been considered as a potential therapeutic target for the treatment of AD.

## Subunits of the *γ*-secretase complex and their assembly

“γ-secretase” was first used to describe the proteolytic activity that cleaves APP within the transmembrane (TM) domain (Haass and Selkoe, 1993). The γ-secretase complex has since been characterized as a high molecular weight complex that consists of four essential subunits: PS (including PS1 and PS2), nicastrin, anterior pharynx defective 1 (APH-1), and presenilin enhancer 2 (PEN-2; De Strooper, 2003; Kimberly et al., 2003; Iwatsubo, 2004). Because of its complexity, almost a decade was required to identify and define all of the components of the γ-secretase complex (De Strooper, 2003).

In the early 1990s, linkage analysis was performed in several FAD families, and two AD-related loci were found on chromosome 1 and chromosome 14 (Schellenberg et al., 1992; Levy-Lahad et al., 1995b). Subsequently, independent research groups identified two homologous genes in these two loci: *PSEN1* (encoding PS1) on chromosome 14 and *PSEN2* (encoding PS2) on chromosome 1 (Levy-Lahad et al., 1995a; Rogaev et al., 1995; Sherrington et al., 1995). Until now, more than 150 different AD-causing mutations have been identified in the two PS genes, where most mutations have been found in *PSEN1* (Vetrivel et al., 2006; De Strooper et al., 2012). FAD-associated PS mutations are directly linked to APP processing and are all seen to increase the relative abundance of the more aggregation-prone Aβ42 compared to Aβ40 (Borchelt et al., 1996, 1997; Duff et al., 1996; Scheuner et al., 1996; Citron et al., 1997; Siman et al., 2000; Flood et al., 2002; Haass et al., 2012). In addition, FAD-linked mutations in PS1 may also affect the trafficking and consequent processing of APP. For instance, FAD-linked PS1 variants can significantly reduce budding of APP-containing vesicles from both the endoplasmic reticulum (ER) and trans Golgi network (TGN), resulting in decreased delivery of APP to the cell surface and increased APP amyloidogenic processing for Aβ generation (Cai et al., 2003). PSs are multi-transmembrane proteins with an undetermined number of TM domains (Doan et al., 1996; Kim and Schekman, 2004). However a nine TM topology model with the amino-terminus in the cytosol and the carboxyl-terminus exposed to the luminal/extracellular space appears to be the most likely depiction of PS topology (Laudon et al., 2005; Oh and Turner, 2005). In general, full-length PS is inactive and rapidly removed by proteolytic degradation (Thinakaran et al., 1996; Podlisny et al., 1997; Ratovitski et al., 1997; Capell et al., 1998; Grunberg et al., 1998). Funtional PS requires endoproteolytic cleavage between TM6 and TM7 of nascently produced PS to generate a 27–28 kDa amino-terminal fragment (NTF) and a 16–17 kDa carboxyl-terminal fragment (CTF). PS NTF and CTF bind to form stable and active PS heterodimers at a 1:1 stoichiometry (Thinakaran et al., 1996, 1997; Podlisny et al., 1997; Ratovitski et al., 1997; Capell et al., 1998; Grunberg et al., 1998). PSs contain two highly conserved aspartate residues in TM6 (D257 in PS1 and D263 in PS2) and TM7 (D385 in PS1 and D366 in PS2), which are indispensable for γ-secretase activity (Wolfe et al., 1999). PS1 heterodimers can be bound by transition-state analog inhibitors of γ-secretase (Esler et al., 2000), suggesting that PSs are the crucial catalytic components of γ-secretase (Li et al., 2000; Kimberly et al., 2003); this notion is confirmed by *in vitro* assays (Ahn et al., 2010). Other PS TM domains also mediate PS function. For example, the TM1 of PS1 was shown to function as a subsite for substrate handling during the processive γ-cleavage in the hydrophilic catalytic pore together with TM 6, 7, and 9 (Takagi et al., 2010; Ohki et al., 2014).

Several studies suggested that it is impossible to overexpress functionally active PS, suggesting that additional protein components are required to form mature, stable PS heterodimers (Baumann et al., 1997; Seeger et al., 1997; Thinakaran et al., 1997; Capell et al., 1998; Yu et al., 1998; Li et al., 2000; Culvenor et al., 2004). The first PS cofactor component identified in the γ-secretase complex is nicastrin (named APH-2 in *C. elegans*), which was identified by screening for modifiers of Notch homologs *glp-1* and* lin-12* in* C. elegans* and through immunochemical purification in HEK293 cells (Goutte et al., 2000; Yu et al., 2000). Nicastrin is a 130 kDa type I TM protein that can be highly glycosylated within its ectodomain (Yu et al., 2000; Yang et al., 2002). Nicastrin is considered to be the scaffolding protein within the γ-secretase complex, and its ectodomain is proposed to bind to the free N-terminus of ectodomain-shed substrates, acting as a substrate receptor in γ-secretase (Shah et al., 2005; Dries et al., 2009).

The other two additional γ-secretase components, APH-1 and PEN-2, were identified through genetic screening in *C. elegans* (Francis et al., 2002; Goutte et al., 2002). PEN-2 spans the membrane twice, with N- and C-terminal domains facing the lumen of the ER (Crystal et al., 2003). There is only one PEN-2 homolog in mammals. Analysis of the APH-1 sequence shows that it contains seven potential TM domains, with the N-terminal domain facing the extracellular space and the C-terminal domain facing the cytosol (Fortna et al., 2004). Two APH-1 homologs, APH-1a and APH-1b have been identified in humans (Francis et al., 2002; Goutte et al., 2002), and one additional homolog APH-1c was identified in mice (Hébert et al., 2004). Mammalian APH-1a has at least two splice variants: APH-1aL and APH-1aS. Since APH1a deletion results in lethality that is not seen in APH-1b and APH-1c gene deletion in mice, different APH-1 isoforms may have different functions. Furthermore, accumulation of APP CTF in specific regions of *APH-1bc*^−/−^ mouse brain (which is equivalent to *APH-1b* deficiency in humans) suggests that APH-1b might be important in the production of Aβ (Serneels et al., 2005). A recent study suggested that γ-secretase complex containing APH-1b tends to generate longer Aβ peptides than complexes containing APH-1a (Acx et al., 2014).

Several studies have demonstrated that the four components of γ-secretase cross-regulate each other coordinately. Down-regulation or deficiency of one given component typically destablizes other components and alters their trafficking/maturation (De Strooper, 2003; Iwatsubo, 2004). For example, in the absence of PS1, PEN-2 is sequestered in the ER and cannot be transported to post-ER components where the mature γ-secretase complex resides (Wang et al., 2004). PS deficiency also leads to destabilization of PEN-2 (Steiner et al., 2002; Luo et al., 2003), which is degraded via the proteasome-mediated pathway (Bergman et al., 2004; Crystal et al., 2004). On the other hand, down-regulation of PEN-2 by small interfering RNA results in an accumulation of full-length PS1 and a reduction of PS1 fragments, suggesting that PEN-2 is involved in PS1 endoproteolysis (Luo et al., 2003). Intracellular trafficking and maturation of nicastrin are also PS dependent. In the absence of PS, nicastrin fails to reach the medial Golgi compartment and becomes only partially glycosylated (Leem et al., 2002). Moreover, nicastrin deficiency reduces the levels of APH-1, PEN-2 and PS1 fragments, and affects their intracellular trafficking (Li et al., 2003a,b; Zhang et al., 2005). Similarly, in APH-1a knockout cells, the levels of nicastrin, PEN-2 and PS1 fragments are reduced (Ma et al., 2005).

The events leading to the formation of a mature γ-secretase complex start from the formation of an initial scaffolding complex composed of APH-1 and nicastrin (LaVoie et al., 2003). The proximal C-terminus of the PS holoprotein then binds to the APH1-nicastrin subcomplex by interacting with the TM domain of nicastrin (Kaether et al., 2004; Jiang et al., 2014). Following PS binding, PEN-2 is incorporated into the complex by interacting with TM4 of PS (Kim and Sisodia, 2005; Watanabe et al., 2005). At the final step, the loop domain between TM6 and TM7 of PS1 is cleaved by endoproteolysis (Fukumori et al., 2010). Alternatively, the APH-1-nicastrin subcomplex may bind directly to a cognate PS1-PEN-2 structure to generate the mature γ-secretase complex (Shirotani et al., 2004; Capell et al., 2005). Several polar residues within APH1 TM domains have been shown to contribute to the assembly and activity of the mature γ-secretase complex (Pardossi-Piquard et al., 2009c), and have been speculated to be involved in γ-secretase substrate presentation (Chen et al., 2010). PEN-2 has also been suggested to be important for stabilizing the complex (Steiner et al., 2002; Prokop et al., 2004, 2005; St George-Hyslop and Fraser, 2012).

Several proteins have been proposed as additional γ-secretase modulatory components, which are not essential for γ-secretase activity. Proteins including CD147 (Zhou et al., 2005), TMP21 (Chen et al., 2006) and γ-secretase activating protein (GSAP; He et al., 2010) have been proposed to selectively modulate Aβ production, but do not affect Notch cleavage. CD147 is a TM glycoprotein, which interacts with all four essential γ-secretase components (Zhou et al., 2005). Downregulation of CD147 increases Aβ production, whereas overexpression of CD147 has no effect on Aβ generation (Zhou et al., 2005). However, another report suggests that CD147 modulates Aβ levels by stimulating the extracellular degradation of Aβ rather than regulating γ-secretase activity directly (Vetrivel et al., 2008). TMP21 is another protein that binds to the γ-secretase complex and regulates γ-cleavage but not ε-cleavage through its TM domain (Chen et al., 2006; Pardossi-Piquard et al., 2009a). However, another study failed to confirm the interaction between TMP21 and γ-secretase, and instead demonstrates that TMP21 influences Aβ generation through APP trafficking (Vetrivel et al., 2007). Recently, a novel GSAP was identified to selectively increase Aβ production through its interaction with both γ-secretase and the APP CTF (He et al., 2010), though interaction between GSAP and APP CTF remains controversial (Hussain et al., 2013). Although the cancer drug Imatinib was reported to also reduce Aβ levels and tau phosphorylation in an AD mouse model by modulating γ-secretase activity and GSAP levels (Chu et al., 2014), these effects could not be reproduced by other groups. Rather, additional studies found that Imatinib had no effect on blood Aβ42 levels in human cancer patients and Aβ production in rats and cell models (Hussain et al., 2013; Olsson et al., 2014).

## Structural characterization of the *γ*-secretase complex

The γ-secretase complex has a molecular weight of approximately 170 kDa, with an additional 30–70 kDa derived from nicastrin glycosylation (Schedin-Weiss et al., 2014), reaching a total size of about 230 kDa with 19 TMs. Structural characterization of the γ-secretase complex is very important for understanding how it recognizes and processes membrane-embedded substrates. However, clarifying details of the γ-secretase structure has gone through a long journey, mainly due to the challenge of expression and purification of an intact γ-secretase complex. The structural information of the γ-secretase complex has been primarily obtained by electron microscopy analysis with a maximum resolution of 12 Å (Lazarov et al., 2006; Ogura et al., 2006; Osenkowski et al., 2009; Renzi et al., 2011; Li et al., 2014), revealing a globular structure with several extracellular domains, three water-accessible cavities, and a potential substrate-binding surface groove in the TM region (Osenkowski et al., 2009). The first solution-state structure of human PS1 CTF was determined by nuclear magnetic resonance, demonstrating that PS1 CTF traverses the membrane three times (Sobhanifar et al., 2010), which is consistent with the widely accepted nine TM structure of PS1 (Laudon et al., 2005; Oh and Turner, 2005). Crystal structure of an archaeal PS homolog also reveals a nine TM topology, with two catalytic aspartate residues located on the cytoplasmic side of TM6 and TM7, and two potential routes for substrate entry (Li et al., 2013).

Recently a three-dimensional structure of the intact human γ-secretase complex was determined by cryo-electronmicroscopy with a resolution of 4.5 Å. The overall structural model comprises a horseshoe-shaped structure with 19 TMs and a bilobed ectodomain representing nicastrin (Lu et al., 2014). Although the resolution is still insufficient to observe atomic details, it was a pioneering step to survey the complete architecture of the γ-secretase complex. The current speculative model suggests that PS1 and PEN-2 are located to the “thick” end of the horseshoe shape, whereas APH-1 and nicastrin are located toward the “thin” end (Lu et al., 2014; Wolfe and Selkoe, 2014). Most recently, results from the same lab presented a crystal structure of nicastrin at 1.95 Å resolution, which is the first atomic-resolution structure for a γ-secretase component (Xie et al., 2014). The extracellular domain of nicastrin contains a large lobe and a small lobe. The large lobe of nicastrin, thought to be responsible for substrate recognition, associates with the small lobe through a hydrophobic pivot at the center (Xie et al., 2014). Based on this new model, nicastrin, APH-1 and PS CTF are likely to be located at the “thick” end of the horseshoe shape, whereas PEN-2 and PS NTF are located toward the “thin” end (Bolduc and Wolfe, 2014; Lu et al., 2014; Xie et al., 2014). Further work is required to elucidate structural details of other γ-secretase components at the atomic level.

## Biological functions of *γ*-secretase

γ-secretase belongs to the family of intramembrane cleaving proteases (i-CLiPs), which includes the presenilin family of aspartyl proteases, the zinc metalloprotease site-2 protease family and the rhomboid family of serine proteases. All i-CLiPs enzymatically cleave their substrates within the plane of the lipid bilayer in a process termed regulated intramembrane proteolysis (Brown et al., 2000; Kopan and Ilagan, 2004). γ-secretase is mainly involved in intramembranous proteolysis of type I membrane proteins. It cleaves numerous functionally important proteins, such as APP (De Strooper et al., 1998), Notch (De Strooper et al., 1999), E-cadherin (Marambaud et al., 2002), ErbB4 (Ni et al., 2001), CD44 (Lammich et al., 2002), tyrosinase (Wang et al., 2006), TREM2 (Wunderlich et al., 2013) and Alcadein (Hata et al., 2012) among others, suggesting the participation of γ-secretase in a vast range of biological activities (Haapasalo and Kovacs, 2011). The best-studied γ-secretase substrates are APP for its roles in AD, and Notch for its importance in development and cell fate determination (Kopan and Ilagan, 2009; Andersson et al., 2011).

During Notch cleavage, γ-secretase releases a Notch intracellular domain (NICD) within the cytosol. NICD can translocate into the nucleus and regulate gene transcription (Kopan et al., 1996; Schroeter et al., 1998). Notch signaling plays a critical role in short-range cell-cell communication during development, as it controls cell fate by regulating cell proliferation, survival, positioning and differentiation (Kopan and Ilagan, 2009; Andersson et al., 2011). Altered expression of Notch target genes such as hairy and enhancer of split (HES) family leads to severe developmental defects. Ablation of γ-secretase by PS1 (Donoviel et al., 1999), nicastrin (Li et al., 2003a,b) and APH-1 (Ma et al., 2005) gene deletion results in embryonic lethality in mice due to ablation of the Notch pathway. Conditional PS1 deletion in various tissues also causes defects associated with Notch pathway, such as defective T- and B-cell differentiation (Doerfler et al., 2001; Hadland et al., 2001; Qyang et al., 2004; Tournoy et al., 2004; Wong et al., 2004), bloody diarrhea as a consequence of gastrointestinal toxicity (Searfoss et al., 2003; Wong et al., 2004; van Es et al., 2005), skin and hair defects (Xia et al., 2001; Tournoy et al., 2004), and depletion of neural progenitor cells accompanied by severe morphological defects and hemorrhages in the developing brain (Kim and Shen, 2008).

APP is initially cleaved by α- or β-secretase, and the remaining membrane-bound C-terminal fragments of APP (APP αCTF and βCTF) are further cleaved by γ-secretase to generate p83 or Aβ, respectively. The p83 fragment is rapidly degraded and widely believed to have negligible function, whereas Aβ is neurotoxic (Selkoe, 2001; Zhang et al., 2011; Proctor et al., 2012; Slowik et al., 2012; Youmans et al., 2012; Chen et al., 2013; Rosén et al., 2013). In addition to releasing Aβ40 and Aβ42, γ-secretase cleavage also generates Aβ46 (ζ-site) (Zhao et al., 2004, 2007) and Aβ49 (ε-site) (Sastre et al., 2001; Weidemann et al., 2002). The existence of different Aβ species, including the shorter Aβ38 fragments suggests that γ-secretase cleaves APP in a sequential manner, first at the ε-site, followed by at the ζ-site, the γ-site, and possibly other sites (Takami et al., 2009; Okochi et al., 2013).

In addition to generating Aβ, γ-secretase cleavage of APP also generates an APP intracellular domain (AICD) within the cell. Similar to NICD, we and others have found that AICD also possess transcriptional transactivation activity and can regulate the transcription of multiple genes including APP, GSK-3b, KAI1, neprilysin, BACE1, p53, EGFR, and LRP1 (Baek et al., 2002; Kim et al., 2003; von Rotz et al., 2004; Pardossi-Piquard et al., 2005; Liu et al., 2007; Zhang et al., 2007). In addition, free AICD can induce apoptosis and may play a role in sensitizing neurons to toxic stimuli (Kinoshita et al., 2002; Giliberto et al., 2008).

## Alternative non-proteolytic functions for *γ*-secretase components

Although PS (Donoviel et al., 1999), nicastrin (Li et al., 2003a,b), or APH-1 (Ma et al., 2005) gene deletion in mice results in lethality and abnormal embryonic phenotypes which resemble that of Notch null mice (Swiatek et al., 1994; Conlon et al., 1995; Huppert et al., 2000), specific phenotypes among different gene deletion strains are not identical, implying that each of these γ-secretase components may have its own unique physiological functions in addition to the γ-secretase activity.

PSs have been thoroughly studied for decades and has been associated with multiple functions, including calcium homeostasis, neurite outgrowth, apoptosis, autophagy, synaptic function, and tumorigenesis (Sisodia et al., 1999; Leem et al., 2002; Thinakaran and Parent, 2004; Kang et al., 2005; Lee et al., 2010; Torres et al., 2012; Bezprozvanny and Hiesinger, 2013; Eimer and Vassar, 2013; Veeraraghavalu et al., 2013; Wang et al., 2014). Several FAD mutations in PSs result in enhanced calcium release via inositol 1,4,5-trisphosphate receptors (IsnP3R) and the ryanodine receptors (RyR) receptors (Cheung et al., 2008; Hayrapetyan et al., 2008; Bezprozvanny and Hiesinger, 2013; Del Prete et al., 2014). PSs also function as passive ER calcium leak channels, and some FAD mutations in PSs disrupt the ER calcium leak function, resulting in elevated ER calcium levels and impaired store-operated calcium entry (Tu et al., 2006; Zhang et al., 2010; Bezprozvanny and Hiesinger, 2013). Moreover, autophagic/lysosomal deficits found in neurons of PS1 deficient mice indicate an essential role of PS1 in lysosomal-dependent proteolysis (Lee et al., 2010). Some studies reported that PSs could participate in neurotransmitter release and regulate synaptic scaling independent of γ-secretase activity (Zhang et al., 2009; Pratt et al., 2011).

Age-related neuronal and synaptic loss and synaptic plasticity deficits in nicastrin conditional knockout mice demonstrates essential roles of nicastrin in regulation of learning and memory and the maintenance of neuronal survival in the brain (Tabuchi et al., 2009; Lee et al., 2014). Furthermore, nicastrin is found to control cell death via Akt and p53-dependent pathways at the post-transcriptional level in a γ-secretase activity-independent manner (Pardossi-Piquard et al., 2009b). APH-1 and PEN2 are also shown to trigger an anti-apoptotic response by lowering p53-dependent control of caspase-3 (Dunys et al., 2007).

## *γ*-secretase as a therapeutic target for AD

γ-secretase is an attractive therapeutic target for AD due to its essential role in the generation of Aβ. Early drug discovery efforts focused on the development of γ-secretase inhibitors (GSIs). However, general inhibition of γ-secretase may potentially result in severe consequences by interfering with other physiological and developmental processes such as its involvement in proteolysis of non-AD components including Notch (Wong et al., 2004; Haapasalo and Kovacs, 2011; Imbimbo et al., 2011; Schor, 2011; Tamayev and D’Adamio, 2012). In a phase III clinical trial of the GSI semagacestat, it was found that semagacestat not only had no effect on improving cognitive status, but also was associated with more adverse events including skin cancers and infections, compared to placebo controls (Doody et al., 2013).

Therefore, the drug discovery efforts have shifted to the development of γ-secretase modulators (GSMs), which are γ-secretase targeting compounds that alter Aβ production without significantly lowering the normal physiological function of Notch and other substrates(Crump et al., 2013). A subset of nonsteroidal anti-inflammatory drugs (NSAIDs) was the first GSM compounds identified (Weggen et al., 2001). R-flurbiprofen (or Tarenflurbil), a single enantiomer of a clinically approved racemic NSAID, had showed some efficacy in a phase II clinical trial with a subgroup of patients suffering from mild AD (Wilcock et al., 2008). However, R-flurbiprofen did not show significant improvement compared to placebo controls during phase III clinical trials (Green et al., 2009). The first generation of GSM compounds demonstrate limited pharmacological potential due to low potency and undesired neuropharmacokinetic properties, while second generation GSMs such as E2012 and EVP-0015962 show improved potency and brain availability and encouraging preclinical profiles in recent years (Oehlrich et al., 2011; Pettersson et al., 2011, 2013). Second generation GSMs can be generally divided into acid GSMs, non-acid GSMs and natural product derived GSMs (Crump et al., 2013; Golde et al., 2013). Acid GSMs, including GSM-1 and its analogs (GSM-2 and GSM-10h) and EVP-0015962 usually reduce Aβ42 and increase Aβ38 levels (Page et al., 2008; Hawkins et al., 2011; Mitani et al., 2012; Rogers et al., 2012). E2012, the first non-acid GSM to enter clinical development, lowers Aβ42 and Aβ40 and raises Aβ37 and Aβ38 levels (Portelius et al., 2010; Borgegard et al., 2012; Crump et al., 2013). Recently identified natural product derived GSMs appear to be unusual as they decrease both Aβ42 and Aβ38 (Hubbs et al., 2012; Loureiro et al., 2013).

## Concluding remarks

The γ-secretase complex plays crucial roles in various physiological processes. Because of the importance of γ-secretase in Aβ generation, γ-secretase has been targeted for AD drug development, but with little success so far due to the complexity of its structural organization and the varied nature of its multiple substrates. A better understanding of the structure-function relationship of γ-secretase will help in developing modulators which limit cleavage of other important physiological γ-secretase substrates for use in AD therapy.

## Conflict of interest statement

The authors declare that the research was conducted in the absence of any commercial or financial relationships that could be construed as a potential conflict of interest.

## References

[B1] Acx H. Chávez-Gutiérrez L. Serneels L. Lismont S. Benurwar M. Elad N.. (2014). Signature amyloid beta profiles are produced by different gamma-secretase complexes. J. Biol. Chem. 289, 4346–4355. 10.1074/jbc.m113.530907 24338474 PMC3924297

[B2] Ahn K. Shelton C. C. Tian Y. Zhang X. Gilchrist M. L. Sisodia S. S.. (2010). Activation and intrinsic gamma-secretase activity of presenilin 1. Proc. Natl. Acad. Sci. U S A 107, 21435–21440. 10.1073/pnas.1013246107 21115843 PMC3003001

[B3] Andersson E. R. Sandberg R. Lendahl U. (2011). Notch signaling: simplicity in design, versatility in function. Development 138, 3593–3612. 10.1242/dev.063610 21828089

[B4] Baek S. H. Ohgi K. A. Rose D. W. Koo E. H. Glass C. K. Rosenfeld M. G. (2002). Exchange of N-CoR corepressor and Tip60 coactivator complexes links gene expression by NF-kappaB and beta-amyloid precursor protein. Cell 110, 55–67. 10.1016/s0092-8674(02)00809-7 12150997

[B5] Baumann K. Paganetti P. A. Sturchler-Pierrat C. Wong C. Hartmann H. Cescato R.. (1997). Distinct processing of endogenous and overexpressed recombinant presenilin 1. Neurobiol. Aging 18, 181–189. 10.1016/s0197-4580(97)00004-3 9258895

[B6] Bergman A. Hansson E. M. Pursglove S. E. Farmery M. R. Lannfelt L. Lendahl U.. (2004). Pen-2 is sequestered in the endoplasmic reticulum and subjected to ubiquitylation and proteasome-mediated degradation in the absence of presenilin. J. Biol. Chem. 279, 16744–16753. 10.1074/jbc.m313999200 14724271

[B7] Bezprozvanny I. Hiesinger P. R. (2013). The synaptic maintenance problem: membrane recycling, Ca2+ homeostasis and late onset degeneration. Mol. Neurodegener. 8:23. 10.1186/1750-1326-8-23 23829673 PMC3708831

[B8] Bolduc D. M. Wolfe M. S. (2014). Structure of nicastrin unveils secrets of gamma-secretase. Proc. Natl. Acad. Sci. U S A 111, 14643–14644. 10.1073/pnas.1416637111 25267656 PMC4205669

[B9] Borchelt D. R. Ratovitski T. van Lare J. Lee M. K. Gonzales V. Jenkins N. A.. (1997). Accelerated amyloid deposition in the brains of transgenic mice coexpressing mutant presenilin 1 and amyloid precursor proteins. Neuron 19, 939–945. 10.1016/s0896-6273(00)80974-5 9354339

[B10] Borchelt D. R. Thinakaran G. Eckman C. B. Lee M. K. Davenport F. Ratovitsky T.. (1996). Familial Alzheimer’s disease-linked presenilin 1 variants elevate Abeta1–42/1–40 ratio in vitro and in vivo. Neuron 17, 1005–1013. 10.1016/s0896-6273(00)80230-5 8938131

[B11] Borgegard T. Juréus A. Olsson F. Rosqvist S. Sabirsh A. Rotticci D.. (2012). First and second generation gamma-secretase modulators (GSMs) modulate amyloid-beta (Abeta) peptide production through different mechanisms. J. Biol. Chem. 287, 11810–11819. 10.1074/jbc.m111.305227 22334705 PMC3320929

[B12] Brown M. S. Ye J. Rawson R. B. Goldstein J. L. (2000). Regulated intramembrane proteolysis: a control mechanism conserved from bacteria to humans. Cell 100, 391–398. 10.1016/s0092-8674(00)80675-3 10693756

[B13] Cai D. Leem J. Y. Greenfield J. P. Wang P. Kim B. S. Wang R.. (2003). Presenilin-1 regulates intracellular trafficking and cell surface delivery of beta-amyloid precursor protein. J. Biol. Chem. 278, 3446–3454. 10.1074/jbc.m209065200 12435726

[B14] Capell A. Beher D. Prokop S. Steiner H. Kaether C. Shearman M. S.. (2005). Gamma-secretase complex assembly within the early secretory pathway. J. Biol. Chem. 280, 6471–6478. 10.1074/jbc.m409106200 15591316

[B15] Capell A. Grünberg J. Pesold B. Diehlmann A. Citron M. Nixon R.. (1998). The proteolytic fragments of the Alzheimer’s disease-associated presenilin-1 form heterodimers and occur as a 100–150-kDa molecular mass complex. J. Biol. Chem. 273, 3205–3211. 10.1074/jbc.273.6.3205 9452432

[B16] Chami L. Checler F. (2012). BACE1 is at the crossroad of a toxic vicious cycle involving cellular stress and beta-amyloid production in Alzheimer’s disease. Mol. Neurodegener. 7:52. 10.1186/1750-1326-7-52 23039869 PMC3507664

[B17] Chen A. C. Guo L. Y. Ostaszewski B. L. Selkoe D. J. LaVoie M. J. (2010). Aph-1 associates directly with full-length and C-terminal fragments of gamma-secretase substrates. J. Biol. Chem. 285, 11378–11391. 10.1074/jbc.m109.088815 20145246 PMC2857016

[B18] Chen F. Hasegawa H. Schmitt-Ulms G. Kawarai T. Bohm C. Katayama T.. (2006). TMP21 is a presenilin complex component that modulates gamma-secretase but not epsilon-secretase activity. Nature 440, 1208–1212. 10.1038/nature04667 16641999

[B19] Chen G. J. Xiong Z. Yan Z. (2013). Abeta impairs nicotinic regulation of inhibitory synaptic transmission and interneuron excitability in prefrontal cortex. Mol. Neurodegener. 8:3. 10.1186/1750-1326-8-3 23327202 PMC3610117

[B20] Cheung K. H. Shineman D. Müller M. Cárdenas C. Mei L. Yang J.. (2008). Mechanism of Ca2+ disruption in Alzheimer’s disease by presenilin regulation of InsP3 receptor channel gating. Neuron 58, 871–883. 10.1016/j.neuron.2008.04.015 18579078 PMC2495086

[B21] Chu J. Lauretti E. Craige C. P. Pratico D. (2014). Pharmacological modulation of GSAP reduces amyloid-beta levels and tau phosphorylation in a mouse model of Alzheimer’s disease with plaques and tangles. J. Alzheimers Dis. 41, 729–737. 10.3233/JAD-140105 24662099

[B22] Citron M. Westaway D. Xia W. Carlson G. Diehl T. Levesque G.. (1997). Mutant presenilins of Alzheimer’s disease increase production of 42-residue amyloid beta-protein in both transfected cells and transgenic mice. Nat. Med. 3, 67–72. 10.1038/nm0197-67 8986743

[B23] Conlon R. A. Reaume A. G. Rossant J. (1995). Notch1 is required for the coordinate segmentation of somites. Development 121, 1533–1545. 7789282 10.1242/dev.121.5.1533

[B24] Crump C. J. Johnson D. S. Li Y. M. (2013). Development and mechanism of gamma-secretase modulators for Alzheimer’s disease. Biochemistry 52, 3197–3216. 10.1021/bi400377p 23614767 PMC3796170

[B25] Crystal A. S. Morais V. A. Fortna R. R. Carlin D. Pierson T. C. Wilson C. A.. (2004). Presenilin modulates Pen-2 levels posttranslationally by protecting it from proteasomal degradation. Biochemistry 43, 3555–3563. 10.1021/bi0361214 15035625

[B26] Crystal A. S. Morais V. A. Pierson T. C. Pijak D. S. Carlin D. Lee V. M.. (2003). Membrane topology of gamma-secretase component PEN-2. J. Biol. Chem. 278, 20117–20123. 10.1074/jbc.m213107200 12639958

[B27] Culvenor J. G. Ilaya N. T. Ryan M. T. Canterford L. Hoke D. E. Williamson N. A.. (2004). Characterization of presenilin complexes from mouse and human brain using Blue Native gel electrophoresis reveals high expression in embryonic brain and minimal change in complex mobility with pathogenic presenilin mutations. Eur. J. Biochem. 271, 375–385. 10.1046/j.1432-1033.2003.03936.x 14717705

[B32] Del Prete D. Checler F. Chami M. (2014). Ryanodine receptors: physiological function and deregulation in Alzheimer disease. Mol. Neurodegener. 9:21. 10.1186/1750-1326-9-21 24902695 PMC4063224

[B28] De Strooper B. (2003). Aph-1, Pen-2 and Nicastrin with Presenilin generate an active gamma-Secretase complex. Neuron 38, 9–12. 10.1016/s0896-6273(03)00205-8 12691659

[B29] De Strooper B. Annaert W. Cupers P. Saftig P. Craessaerts K. Mumm J. S.. (1999). A presenilin-1-dependent gamma-secretase-like protease mediates release of Notch intracellular domain. Nature 398, 518–522. 10.1038/19083 10206645

[B30] De Strooper B. Iwatsubo T. Wolfe M. S. (2012). Presenilins and gamma-secretase: structure, function and role in Alzheimer disease. Cold Spring Harb. Perspect. Med. 2:a006304. 10.1101/cshperspect.a006304 22315713 PMC3253024

[B31] De Strooper B. Saftig P. Craessaerts K. Vanderstichele H. Guhde G. Annaert W.. (1998). Deficiency of presenilin-1 inhibits the normal cleavage of amyloid precursor protein. Nature 391, 387–390. 10.1038/34910 9450754

[B33] Doan A. Thinakaran G. Borchelt D. R. Slunt H. H. Ratovitsky T. Podlisny M.. (1996). Protein topology of presenilin 1. Neuron 17, 1023–1030. 10.1016/S0896-6273(00)80232-9 8938133

[B34] Doerfler P. Shearman M. S. Perlmutter R. M. (2001). Presenilin-dependent gamma-secretase activity modulates thymocyte development. Proc. Natl. Acad. Sci. U S A 98, 9312–9317. 10.1073/pnas.161102498 11470902 PMC55417

[B35] Donoviel D. B. Hadjantonakis A. K. Ikeda M. Zheng H. Hyslop P. S. Bernstein A. (1999). Mice lacking both presenilin genes exhibit early embryonic patterning defects. Genes Dev. 13, 2801–2810. 10.1101/gad.13.21.2801 10557208 PMC317124

[B36] Doody R. S. Raman R. Farlow M. Iwatsubo T. Vellas B. Joffe S.. (2013). A phase 3 trial of semagacestat for treatment of Alzheimer’s disease. N. Engl. J. Med. 369, 341–350. 10.1056/NEJMoa1210951 23883379

[B37] Dries D. R. Shah S. Han Y. H. Yu C. Yu S. Shearman M. S.. (2009). Glu-333 of nicastrin directly participates in gamma-secretase activity. J. Biol. Chem. 284, 29714–29724. 10.1074/jbc.m109.038737 19729449 PMC2785603

[B38] Duff K. Eckman C. Zehr C. Yu X. Prada C. M. Perez-tur J.. (1996). Increased amyloid-beta42(43) in brains of mice expressing mutant presenilin 1. Nature 383, 710–713. 10.1038/383710a0 8878479

[B39] Dunys J. Kawarai T. Sevalle J. Dolcini V. George-Hyslop P. S. Da Costa C. A.. (2007). p53-Dependent Aph-1 and Pen-2 anti-apoptotic phenotype requires the integrity of the gamma-secretase complex but is independent of its activity. J. Biol. Chem. 282, 10516–10525. 10.1074/jbc.m611572200 17276981

[B40] Eimer W. A. Vassar R. (2013). Neuron loss in the 5XFAD mouse model of Alzheimer’s disease correlates with intraneuronal Abeta42 accumulation and Caspase-3 activation. Mol. Neurodegener. 8:2. 10.1186/1750-1326-8-2 23316765 PMC3552866

[B41] Esler W. P. Kimberly W. T. Ostaszewski B. L. Diehl T. S. Moore C. L. Tsai J. Y.. (2000). Transition-state analogue inhibitors of gamma-secretase bind directly to presenilin-1. Nat. Cell Biol. 2, 428–434. 10.1038/35017062 10878808

[B42] Flood D. G. Reaume A. G. Dorfman K. S. Lin Y. G. Lang D. M. Trusko S. P.. (2002). FAD mutant PS-1 gene-targeted mice: increased A beta 42 and A beta deposition without APP overproduction. Neurobiol. Aging 23, 335–348. 10.1016/s0197-4580(01)00330-x 11959395

[B43] Fortna R. R. Crystal A. S. Morais V. A. Pijak D. S. Lee V. M. Doms R. W. (2004). Membrane topology and nicastrin-enhanced endoproteolysis of APH-1, a component of the gamma-secretase complex. J. Biol. Chem. 279, 3685–3693. 10.1074/jbc.m310505200 14593096

[B44] Francis R. McGrath G. Zhang J. Ruddy D. A. Sym M. Apfeld J.. (2002). aph-1 and pen-2 are required for Notch pathway signaling, gamma-secretase cleavage of betaAPP and presenilin protein accumulation. Dev. Cell 3, 85–97. 10.1016/S1534-5807(02)00189-2 12110170

[B45] Fukumori A. Fluhrer R. Steiner H. Haass C. (2010). Three-amino acid spacing of presenilin endoproteolysis suggests a general stepwise cleavage of gamma-secretase-mediated intramembrane proteolysis. J. Neurosci. 30, 7853–7862. 10.1523/jneurosci.1443-10.2010 20534834 PMC6632680

[B46] Giliberto L. Zhou D. Weldon R. Tamagno E. De Luca P. Tabaton M.. (2008). Evidence that the Amyloid beta Precursor Protein-intracellular domain lowers the stress threshold of neurons and has a “regulated” transcriptional role. Mol. Neurodegener. 3:12. 10.1186/1750-1326-3-12 18764939 PMC2538519

[B47] Glenner G. G. Wong C. W. (1984). Alzheimer’s disease: initial report of the purification and characterization of a novel cerebrovascular amyloid protein. Biochem. Biophys. Res. Commun. 120, 885–890. 10.1016/S0006-291X(84)80190-4 6375662

[B48] Goate A. Chartier-Harlin M. C. Mullan M. Brown J. Crawford F. Fidani L.. (1991). Segregation of a missense mutation in the amyloid precursor protein gene with familial Alzheimer’s disease. Nature 349, 704–706. 10.1038/349704a0 1671712

[B49] Golde T. E. (2005). The Abeta hypothesis: leading us to rationally-designed therapeutic strategies for the treatment or prevention of Alzheimer disease. Brain Pathol. 15, 84–87. 10.1111/j.1750-3639.2005.tb00104.x 15779241 PMC8095797

[B50] Golde T. E. Koo E. H. Felsenstein K. M. Osborne B. A. Miele L. (2013). gamma-Secretase inhibitors and modulators. Biochim. Biophys. Acta 1828, 2898–2907. 10.1016/j.bbamem.2013.06.005 23791707 PMC3857966

[B51] Goutte C. Hepler W. Mickey K. M. Priess J. R. (2000). aph-2 encodes a novel extracellular protein required for GLP-1-mediated signaling. Development 127, 2481–2492. 10804188 10.1242/dev.127.11.2481

[B52] Goutte C. Tsunozaki M. Hale V. A. Priess J. R. (2002). APH-1 is a multipass membrane protein essential for the Notch signaling pathway in Caenorhabditis elegans embryos. Proc. Natl. Acad. Sci. U S A 99, 775–779. 10.1073/pnas.022523499 11792846 PMC117381

[B53] Green R. C. Schneider L. S. Amato D. A. Beelen A. P. Wilcock G. Swabb E. A.. (2009). Effect of tarenflurbil on cognitive decline and activities of daily living in patients with mild Alzheimer disease: a randomized controlled trial. JAMA 302, 2557–2564. 10.1001/jama.2009.1866 20009055 PMC2902875

[B54] Greenfield J. P. Gross R. S. Gouras G. K. Xu H. (2000). Cellular and molecular basis of beta-amyloid precursor protein metabolism. Front. Biosci. 5, D72–D83. 10.2741/greenfield 10702374

[B55] Grunberg J. Walter J. Loetscher H. Deuschle U. Jacobsen H. Haass C. (1998). Alzheimer’s disease associated presenilin-1 holoprotein and its 18–20 kDa C-terminal fragment are death substrates for proteases of the caspase family. Biochemistry 37, 2263–2270. 10.1021/bi972106l 9485372

[B56] Haapasalo A. Kovacs D. M. (2011). The many substrates of presenilin/gamma-secretase. J. Alzheimers Dis. 25, 3–28. 10.3233/JAD-2011-101065 21335653 PMC3281584

[B57] Haass C. De Strooper B. (1999). The presenilins in Alzheimer’s disease–proteolysis holds the key. Science 286, 916–919. 10.1126/science.286.5441.916 10542139

[B58] Haass C. Kaether C. Thinakaran G. Sisodia S. (2012). Trafficking and proteolytic processing of APP. Cold Spring Harb. Perspect. Med. 2:a006270. 10.1101/cshperspect.a006270 22553493 PMC3331683

[B59] Haass C. Selkoe D. J. (1993). Cellular processing of beta-amyloid precursor protein and the genesis of amyloid beta-peptide. Cell 75, 1039–1042. 10.1016/0092-8674(93)90312-e 8261505

[B60] Hadland B. K. Manley N. R. Su D. Longmore G. D. Moore C. L. Wolfe M. S.. (2001). Gamma -secretase inhibitors repress thymocyte development. Proc. Natl. Acad. Sci. U S A 98, 7487–7491. 10.1073/pnas.131202798 11416218 PMC34695

[B61] Hata S. Taniguchi M. Piao Y. Ikeuchi T. Fagan A. M. Holtzman D. M.. (2012). Multiple gamma-secretase product peptides are coordinately increased in concentration in the cerebrospinal fluid of a subpopulation of sporadic Alzheimer’s disease subjects. Mol. Neurodegener. 7:16. 10.1186/1750-1326-7-16 22534039 PMC3422204

[B62] Hawkins J. Harrison D. C. Ahmed S. Davis R. P. Chapman T. Marshall I.. (2011). Dynamics of Abeta42 reduction in plasma, CSF and brain of rats treated with the gamma-secretase modulator, GSM-10h. Neurodegener. Dis. 8, 455–464. 10.1159/000324511 21389687

[B63] Hayrapetyan V. Rybalchenko V. Rybalchenko N. Koulen P. (2008). The N-terminus of presenilin-2 increases single channel activity of brain ryanodine receptors through direct protein-protein interaction. Cell Calcium 44, 507–518. 10.1016/j.ceca.2008.03.004 18440065

[B64] He G. Luo W. Li P. Remmers C. Netzer W. J. Hendrick J.. (2010). Gamma-secretase activating protein is a therapeutic target for Alzheimer’s disease. Nature 467, 95–98. 10.1038/nature09325 20811458 PMC2936959

[B65] Hébert S. S. Serneels L. Dejaegere T. Horré K. Dabrowski M. Baert V.. (2004). Coordinated and widespread expression of gamma-secretase in vivo: evidence for size and molecular heterogeneity. Neurobiol. Dis. 17, 260–272. 10.1016/j.nbd.2004.08.002 15474363

[B66] Hubbs J. L. Fuller N. O. Austin W. F. Shen R. Creaser S. P. McKee T. D.. (2012). Optimization of a natural product-based class of gamma-secretase modulators. J. Med. Chem. 55, 9270–9282. 10.1021/jm300976b 23030762

[B67] Huppert S. S. Le A. Schroeter E. H. Mumm J. S. Saxena M. T. Milner L. A.. (2000). Embryonic lethality in mice homozygous for a processing-deficient allele of Notch1. Nature 405, 966–970. 10.1038/35016111 10879540

[B68] Hussain I. Fabrègue J. Anderes L. Ousson S. Borlat F. Eligert V.. (2013). The role of gamma-secretase activating protein (GSAP) and imatinib in the regulation of gamma-secretase activity and amyloid-beta generation. J. Biol. Chem. 288, 2521–2531. 10.1074/jbc.m112.370924 23209290 PMC3554920

[B69] Imbimbo B. P. Panza F. Frisardi V. Solfrizzi V. D’onofrio G. Logroscino G.. (2011). Therapeutic intervention for Alzheimer’s disease with gamma-secretase inhibitors: still a viable option? Expert Opin. Investig. Drugs 20, 325–341. 10.1517/13543784.2011.550572 21222550

[B70] Iwatsubo T. (2004). The gamma-secretase complex: machinery for intramembrane proteolysis. Curr. Opin. Neurobiol. 14, 379–383. 10.1016/s0959-4388(04)00077-7 15194119

[B71] Jiang S. Li Y. Zhang X. Bu G. Xu H. Zhang Y. W. (2014). Trafficking regulation of proteins in Alzheimer’s disease. Mol. Neurodegener. 9:6. 10.1186/1750-1326-9-6 24410826 PMC3891995

[B72] Kaether C. Capell A. Edbauer D. Winkler E. Novak B. Steiner H.. (2004). The presenilin C-terminus is required for ER-retention, nicastrin-binding and gamma-secretase activity. EMBO J. 23, 4738–4748. 10.1038/sj.emboj.7600478 15549135 PMC535090

[B73] Kang D. E. Yoon I. S. Repetto E. Busse T. Yermian N. Ie L.. (2005). Presenilins mediate phosphatidylinositol 3-kinase/AKT and ERK activation via select signaling receptors. Selectivity of PS2 in platelet-derived growth factor signaling. J. Biol. Chem. 280, 31537–31547. 10.1074/jbc.m500833200 16014629

[B74] Kim H. S. Kim E. M. Lee J. P. Park C. H. Kim S. Seo J. H.. (2003). C-terminal fragments of amyloid precursor protein exert neurotoxicity by inducing glycogen synthase kinase-3beta expression. FASEB J. 17, 1951–1953. 10.1096/fj.03-0106fje 12923068

[B75] Kim J. Schekman R. (2004). The ins and outs of presenilin 1 membrane topology. Proc. Natl. Acad. Sci. U S A 101, 905–906. 10.1073/pnas.0307297101 14732690 PMC327113

[B76] Kim W. Y. Shen J. (2008). Presenilins are required for maintenance of neural stem cells in the developing brain. Mol. Neurodegener. 3:2. 10.1186/1750-1326-3-2 18182109 PMC2235867

[B77] Kim S. H. Sisodia S. S. (2005). Evidence that the “NF” motif in transmembrane domain 4 of presenilin 1 is critical for binding with PEN-2. J. Biol. Chem. 280, 41953–41966. 10.1074/jbc.m509070200 16234243

[B78] Kimberly W. T. LaVoie M. J. Ostaszewski B. L. Ye W. Wolfe M. S. Selkoe D. J. (2003). Gamma-secretase is a membrane protein complex comprised of presenilin, nicastrin, Aph-1 and Pen-2. Proc. Natl. Acad. Sci. U S A 100, 6382–6387. 10.1073/pnas.1037392100 12740439 PMC164455

[B79] Kinoshita A. Whelan C. M. Berezovska O. Hyman B. T. (2002). The gamma secretase-generated carboxyl-terminal domain of the amyloid precursor protein induces apoptosis via Tip60 in H4 cells. J. Biol. Chem. 277, 28530–28536. 10.1074/jbc.m203372200 12032152

[B80] Kopan R. Ilagan M. X. (2004). Gamma-secretase: proteasome of the membrane? Nat. Rev. Mol. Cell Biol. 5, 499–504. 10.1038/nrm1406 15173829

[B81] Kopan R. Ilagan M. X. (2009). The canonical Notch signaling pathway: unfolding the activation mechanism. Cell 137, 216–233. 10.1016/j.cell.2009.03.045 19379690 PMC2827930

[B82] Kopan R. Schroeter E. H. Weintraub H. Nye J. S. (1996). Signal transduction by activated mNotch: importance of proteolytic processing and its regulation by the extracellular domain. Proc. Natl. Acad. Sci. U S A 93, 1683–1688. 10.1073/pnas.93.4.1683 8643690 PMC40002

[B83] Kumar-Singh S. Theuns J. Van Broeck B. Pirici D. Vennekens K. Corsmit E.. (2006). Mean age-of-onset of familial alzheimer disease caused by presenilin mutations correlates with both increased Abeta42 and decreased Abeta40. Hum. Mutat. 27, 686–695. 10.1002/humu.20336 16752394

[B84] Lammich S. Okochi M. Takeda M. Kaether C. Capell A. Zimmer A. K.. (2002). Presenilin-dependent intramembrane proteolysis of CD44 leads to the liberation of its intracellular domain and the secretion of an Abeta-like peptide. J. Biol. Chem. 277, 44754–44759. 10.1074/jbc.m206872200 12223485

[B85] Laudon H. Hansson E. M. Melén K. Bergman A. Farmery M. R. Winblad B.. (2005). A nine-transmembrane domain topology for presenilin 1. J. Biol. Chem. 280, 35352–35360. 10.1074/jbc.m507217200 16046406

[B86] LaVoie M. J. Fraering P. C. Ostaszewski B. L. Ye W. Kimberly W. T. Wolfe M. S.. (2003). Assembly of the gamma-secretase complex involves early formation of an intermediate subcomplex of Aph-1 and nicastrin. J. Biol. Chem. 278, 37213–37222. 10.1074/jbc.m303941200 12857757

[B87] Lazarov V. K. Fraering P. C. Ye W. Wolfe M. S. Selkoe D. J. Li H. (2006). Electron microscopic structure of purified, active gamma-secretase reveals an aqueous intramembrane chamber and two pores. Proc. Natl. Acad. Sci. U S A 103, 6889–6894. 10.1073/pnas.0602321103 16636269 PMC1458989

[B89] Lee S. H. Sharma M. Südhof T. C. Shen J. (2014). Synaptic function of nicastrin in hippocampal neurons. Proc. Natl. Acad. Sci. U S A 111, 8973–8978. 10.1073/pnas.1408554111 24889619 PMC4066509

[B88] Lee J. H. Yu W. H. Kumar A. Lee S. Mohan P. S. Peterhoff C. M.. (2010). Lysosomal proteolysis and autophagy require presenilin 1 and are disrupted by Alzheimer-related PS1 mutations. Cell 141, 1146–1158. 10.1016/j.cell.2010.05.008 20541250 PMC3647462

[B90] Leem J. Y. Vijayan S. Han P. Cai D. Machura M. Lopes K. O.. (2002). Presenilin 1 is required for maturation and cell surface accumulation of nicastrin. J. Biol. Chem. 277, 19236–19240. 10.1074/jbc.c200148200 11943765

[B91] Levy-Lahad E. Wasco W. Poorkaj P. Romano D. M. Oshima J. Pettingell W. H.. (1995a). Candidate gene for the chromosome 1 familial Alzheimer’s disease locus. Science 269, 973–977. 10.1126/science.7638622 7638622

[B92] Levy-Lahad E. Wijsman E. M. Nemens E. Anderson L. Goddard K. A. Weber J. L.. (1995b). A familial Alzheimer’s disease locus on chromosome 1. Science 269, 970–973. 10.1126/science.7638621 7638621

[B93] Li X. Dang S. Yan C. Gong X. Wang J. Shi Y. (2013). Structure of a presenilin family intramembrane aspartate protease. Nature 493, 56–61. 10.1038/nature11801 23254940

[B94] Li J. Fici G. J. Mao C. A. Myers R. L. Shuang R. Donoho G. P.. (2003a). Positive and negative regulation of the gamma-secretase activity by nicastrin in a murine model. J. Biol. Chem. 278, 33445–33449. 10.1074/jbc.m301288200 12815056

[B95] Li Y. M. Lai M. T. Xu M. Huang Q. Dimuzio-Mower J. Sardana M. K.. (2000). Presenilin 1 is linked with gamma-secretase activity in the detergent solubilized state. Proc. Natl. Acad. Sci. U S A 97, 6138–6143. 10.1073/pnas.110126897 10801983 PMC18571

[B96] Li Y. Lu S. H. Tsai C. J. Bohm C. Qamar S. Dodd R. B.. (2014). Structural interactions between inhibitor and substrate docking sites give insight into mechanisms of human PS1 complexes. Structure 22, 125–135. 10.1016/j.str.2013.09.018 24210759 PMC3887256

[B97] Li T. Ma G. Cai H. Price D. L. Wong P. C. (2003b). Nicastrin is required for assembly of presenilin/gamma-secretase complexes to mediate Notch signaling and for processing and trafficking of beta-amyloid precursor protein in mammals. J. Neurosci. 23, 3272–3277. 12716934 10.1523/JNEUROSCI.23-08-03272.2003 PMC6742329

[B98] Liu Q. Zerbinatti C. V. Zhang J. Hoe H. S. Wang B. Cole S. L.. (2007). Amyloid precursor protein regulates brain apolipoprotein E and cholesterol metabolism through lipoprotein receptor LRP1. Neuron 56, 66–78. 10.1016/j.neuron.2007.08.008 17920016 PMC2045076

[B99] Loureiro R. M. Dumin J. A. McKee T. D. Austin W. F. Fuller N. O. Hubbs J. L.. (2013). Efficacy of SPI-1865, a novel gamma-secretase modulator, in multiple rodent models. Alzheimers Res. Ther. 5:19. 10.1186/alzrt173 23597079 PMC3707052

[B100] Lu P. Bai X. C. Ma D. Xie T. Yan C. Sun L.. (2014). Three-dimensional structure of human gamma-secretase. Nature 512, 166–170. 10.1038/nature13567 25043039 PMC4134323

[B101] Luo W. J. Wang H. Li H. Kim B. S. Shah S. Lee H. J.. (2003). PEN-2 and APH-1 coordinately regulate proteolytic processing of presenilin 1. J. Biol. Chem. 278, 7850–7854. 10.1074/jbc.c200648200 12522139

[B102] Ma G. Li T. Price D. L. Wong P. C. (2005). APH-1a is the principal mammalian APH-1 isoform present in gamma-secretase complexes during embryonic development. J. Neurosci. 25, 192–198. 10.1523/jneurosci.3814-04.2005 15634781 PMC6725209

[B103] Marambaud P. Shioi J. Serban G. Georgakopoulos A. Sarner S. Nagy V.. (2002). A presenilin-1/gamma-secretase cleavage releases the E-cadherin intracellular domain and regulates disassembly of adherens junctions. EMBO J. 21, 1948–1956. 10.1093/emboj/21.8.1948 11953314 PMC125968

[B104] Masters C. L. Simms G. Weinman N. A. Multhaup G. McDonald B. L. Beyreuther K. (1985). Amyloid plaque core protein in Alzheimer disease and Down syndrome. Proc. Natl. Acad. Sci. U S A 82, 4245–4249. 10.1073/pnas.82.12.4245 3159021 PMC397973

[B105] McColl G. Roberts B. R. Pukala T. L. Kenche V. B. Roberts C. M. Link C. D.. (2012). Utility of an improved model of amyloid-beta (Abeta(1)(-)(4)(2)) toxicity in Caenorhabditis elegans for drug screening for Alzheimer’s disease. Mol. Neurodegener. 7:57. 10.1186/1750-1326-7-57 23171715 PMC3519830

[B106] Mitani Y. Yarimizu J. Saita K. Uchino H. Akashiba H. Shitaka Y.. (2012). Differential effects between gamma-secretase inhibitors and modulators on cognitive function in amyloid precursor protein-transgenic and nontransgenic mice. J. Neurosci. 32, 2037–2050. 10.1523/jneurosci.4264-11.2012 22323718 PMC6621706

[B107] Ni C. Y. Murphy M. P. Golde T. E. Carpenter G. (2001). gamma -Secretase cleavage and nuclear localization of ErbB-4 receptor tyrosine kinase. Science 294, 2179–2181. 10.1126/science.1065412 11679632

[B108] Oehlrich D. Berthelot D. J. Gijsen H. J. (2011). gamma-Secretase modulators as potential disease modifying anti-Alzheimer’s drugs. J. Med. Chem. 54, 669–698. 10.1021/jm101168r 21141968

[B109] Ogura T. Mio K. Hayashi I. Miyashita H. Fukuda R. Kopan R.. (2006). Three-dimensional structure of the gamma-secretase complex. Biochem. Biophys. Res. Commun. 343, 525–534. 10.1016/j.bbrc.2006.02.158 16546128

[B110] Oh Y. S. Turner R. J. (2005). Topology of the C-terminal fragment of human presenilin 1. Biochemistry 44, 11821–11828. 10.1021/bi0509494 16128583

[B111] Ohki Y. Shimada N. Tominaga A. Osawa S. Higo T. Yokoshima S.. (2014). Binding of longer Abeta to transmembrane domain 1 of presenilin 1 impacts on Abeta42 generation. Mol. Neurodegener. 9:7. 10.1186/1750-1326-9-7 24410857 PMC3896738

[B112] Okochi M. Tagami S. Yanagida K. Takami M. Kodama T. S. Mori K.. (2013). gamma-secretase modulators and presenilin 1 mutants act differently on presenilin/gamma-secretase function to cleave Abeta42 and Abeta43. Cell Rep. 3, 42–51. 10.1016/j.celrep.2012.11.028 23291095

[B113] Olsson B. Legros L. Guilhot F. Strömberg K. Smith J. Livesey F. J.. (2014). Imatinib treatment and Abeta42 in humans. Alzheimers Dement. 10, S374–S380. 10.3410/f.12445.469930 24331439

[B114] Osenkowski P. Li H. Ye W. Li D. Aeschbach L. Fraering P. C.. (2009). Cryoelectron microscopy structure of purified gamma-secretase at 12 A resolution. J. Mol. Biol. 385, 642–652. 10.1016/j.jmb.2008.10.078 19013469 PMC2830092

[B115] Page R. M. Baumann K. Tomioka M. Pérez-Revuelta B. I. Fukumori A. Jacobsen H.. (2008). Generation of Abeta38 and Abeta42 is independently and differentially affected by familial Alzheimer disease-associated presenilin mutations and gamma-secretase modulation. J. Biol. Chem. 283, 677–683. 10.1074/jbc.m708754200 17962197

[B116] Pardossi-Piquard R. Böhm C. Chen F. Kanemoto S. Checler F. Schmitt-Ulms G.. (2009a). TMP21 transmembrane domain regulates gamma-secretase cleavage. J. Biol. Chem. 284, 28634–28641. 10.1074/jbc.m109.059345 19710022 PMC2781407

[B117] Pardossi-Piquard R. Dunys J. Giaime E. Guillot-Sestier M. V. St George-Hyslop P. Checler F.. (2009b). p53-dependent control of cell death by nicastrin: lack of requirement for presenilin-dependent gamma-secretase complex. J. Neurochem. 109, 225–237. 10.1111/j.1471-4159.2009.05952.x 19187441

[B118] Pardossi-Piquard R. Petit A. Kawarai T. Sunyach C. Alves da Costa C. Vincent B.. (2005). Presenilin-dependent transcriptional control of the Abeta-degrading enzyme neprilysin by intracellular domains of betaAPP and APLP. Neuron 46, 541–554. 10.1016/j.neuron.2005.04.008 15944124

[B119] Pardossi-Piquard R. Yang S. P. Kanemoto S. Gu Y. Chen F. Böhm C.. (2009c). APH1 polar transmembrane residues regulate the assembly and activity of presenilin complexes. J. Biol. Chem. 284, 16298–16307. 10.1074/jbc.m109.000067 19369254 PMC2713549

[B120] Pettersson M. Kauffman G. W. am Ende C. W. Patel N. C. Stiff C. Tran T. P.. (2011). Novel gamma-secretase modulators: a review of patents from 2008 to 2010. Expert Opin. Ther. Pat. 21, 205–226. 10.1517/13543776.2011.547479 21231889

[B121] Pettersson M. Stepan A. F. Kauffman G. W. Johnson D. S. (2013). Novel gamma-secretase modulators for the treatment of Alzheimer’s disease: a review focusing on patents from 2010 to 2012. Expert Opin. Ther. Pat. 23, 1349–1366. 10.1517/13543776.2013.821465 23875696

[B122] Podlisny M. B. Citron M. Amarante P. Sherrington R. Xia W. Zhang J.. (1997). Presenilin proteins undergo heterogeneous endoproteolysis between Thr291 and Ala299 and occur as stable N- and C-terminal fragments in normal and Alzheimer brain tissue. Neurobiol. Dis. 3, 325–337. 10.1006/nbdi.1997.0129 9173929

[B123] Portelius E. Van Broeck B. Andreasson U. Gustavsson M. K. Mercken M. Zetterberg H.. (2010). Acute effect on the Abeta isoform pattern in CSF in response to gamma-secretase modulator and inhibitor treatment in dogs. J. Alzheimers Dis. 21, 1005–1012. 10.3233/JAD-2010-100573 20634579

[B124] Pratt K. G. Zimmerman E. C. Cook D. G. Sullivan J. M. (2011). Presenilin 1 regulates homeostatic synaptic scaling through Akt signaling. Nat. Neurosci. 14, 1112–1114. 10.1038/nn.2893 21841774 PMC3164917

[B125] Proctor C. J. Pienaar I. S. Elson J. L. Kirkwood T. B. (2012). Aggregation, impaired degradation and immunization targeting of amyloid-beta dimers in Alzheimer’s disease: a stochastic modelling approach. Mol. Neurodegener. 7:32. 10.1186/1750-1326-7-32 22748062 PMC3583291

[B126] Prokop S. Haass C. Steiner H. (2005). Length and overall sequence of the PEN-2 C-terminal domain determines its function in the stabilization of presenilin fragments. J. Neurochem. 94, 57–62. 10.1111/j.1471-4159.2005.03165.x 15953349

[B127] Prokop S. Shirotani K. Edbauer D. Haass C. Steiner H. (2004). Requirement of PEN-2 for stabilization of the presenilin N-/C-terminal fragment heterodimer within the gamma-secretase complex. J. Biol. Chem. 279, 23255–23261. 10.1074/jbc.m401789200 15039426

[B128] Qyang Y. Chambers S. M. Wang P. Xia X. Chen X. Goodell M. A.. (2004). Myeloproliferative disease in mice with reduced presenilin gene dosage: effect of gamma-secretase blockage. Biochemistry 43, 5352–5359. 10.1021/bi049826u 15122901

[B129] Ratovitski T. Slunt H. H. Thinakaran G. Price D. L. Sisodia S. S. Borchelt D. R. (1997). Endoproteolytic processing and stabilization of wild-type and mutant presenilin. J. Biol. Chem. 272, 24536–24541. 10.1074/jbc.272.39.24536 9305918

[B130] Renzi F. Zhang X. Rice W. J. Torres-Arancivia C. Gomez-Llorente Y. Diaz R.. (2011). Structure of gamma-secretase and its trimeric pre-activation intermediate by single-particle electron microscopy. J. Biol. Chem. 286, 21440–21449. 10.1074/jbc.m110.193326 21454611 PMC3122203

[B131] Rogaev E. I. Sherrington R. Rogaeva E. A. Levesque G. Ikeda M. Liang Y.. (1995). Familial Alzheimer’s disease in kindreds with missense mutations in a gene on chromosome 1 related to the Alzheimer’s disease type 3 gene. Nature 376, 775–778. 10.1038/376775a0 7651536

[B132] Rogers K. Felsenstein K. M. Hrdlicka L. Tu Z. Albayya F. Lee W.. (2012). Modulation of gamma-secretase by EVP-0015962 reduces amyloid deposition and behavioral deficits in Tg2576 mice. Mol. Neurodegener. 7:61. 10.1186/1750-1326-7-61 23249765 PMC3573960

[B133] Rosén C. Hansson O. Blennow K. Zetterberg H. (2013). Fluid biomarkers in Alzheimer’s disease - current concepts. Mol. Neurodegener. 8:20. 10.1186/1750-1326-8-20 23800368 PMC3691925

[B134] Sastre M. Steiner H. Fuchs K. Capell A. Multhaup G. Condron M. M.. (2001). Presenilin-dependent gamma-secretase processing of beta-amyloid precursor protein at a site corresponding to the S3 cleavage of Notch. EMBO Rep. 2, 835–841. 10.1093/embo-reports/kve180 11520861 PMC1084035

[B135] Schedin-Weiss S. Winblad B. Tjernberg L. O. (2014). The role of protein glycosylation in Alzheimer disease. FEBS J. 281, 46–62. 10.1111/febs.12590 24279329

[B136] Schellenberg G. D. Bird T. D. Wijsman E. M. Orr H. T. Anderson L. Nemens E.. (1992). Genetic linkage evidence for a familial Alzheimer’s disease locus on chromosome 14. Science 258, 668–671. 10.1126/science.1411576 1411576

[B137] Scheuner D. Eckman C. Jensen M. Song X. Citron M. Suzuki N.. (1996). Secreted amyloid beta-protein similar to that in the senile plaques of Alzheimer’s disease is increased in vivo by the presenilin 1 and 2 and APP mutations linked to familial Alzheimer’s disease. Nat. Med. 2, 864–870. 10.1038/nm0896-864 8705854

[B138] Schor N. F. (2011). What the halted phase III gamma-secretase inhibitor trial may (or may not) be telling us. Ann. Neurol. 69, 237–239. 10.1002/ana.22365 21387368

[B139] Schroeter E. H. Kisslinger J. A. Kopan R. (1998). Notch-1 signalling requires ligand-induced proteolytic release of intracellular domain. Nature 393, 382–386. 10.1038/30756 9620803

[B140] Searfoss G. H. Jordan W. H. Calligaro D. O. Galbreath E. J. Schirtzinger L. M. Berridge B. R.. (2003). Adipsin, a biomarker of gastrointestinal toxicity mediated by a functional gamma-secretase inhibitor. J. Biol. Chem. 278, 46107–46116. 10.1074/jbc.m307757200 12949072

[B141] Seeger M. Nordstedt C. Petanceska S. Kovacs D. M. Gouras G. K. Hahne S.. (1997). Evidence for phosphorylation and oligomeric assembly of presenilin 1. Proc. Natl. Acad. Sci. U S A 94, 5090–5094. 10.1073/pnas.94.10.5090 9144195 PMC24636

[B142] Selkoe D. J. (1998). The cell biology of beta-amyloid precursor protein and presenilin in Alzheimer’s disease. Trends Cell Biol. 8, 447–453. 10.1016/S0962-8924(98)01363-4 9854312

[B143] Selkoe D. J. (2001). Alzheimer’s disease results from the cerebral accumulation and cytotoxicity of amyloid beta-protein. J. Alzheimers Dis. 3, 75–80. 12214075 10.3233/jad-2001-3111

[B144] Serneels L. Dejaegere T. Craessaerts K. Horré K. Jorissen E. Tousseyn T.. (2005). Differential contribution of the three Aph1 genes to gamma-secretase activity in vivo. Proc. Natl. Acad. Sci. U S A 102, 1719–1724. 10.1073/pnas.0408901102 15665098 PMC547495

[B145] Shah S. Lee S. F. Tabuchi K. Hao Y. H. Yu C. Laplant Q.. (2005). Nicastrin functions as a gamma-secretase-substrate receptor. Cell 122, 435–447. 10.1016/j.cell.2005.05.022 16096062

[B146] Sherrington R. Rogaev E. I. Liang Y. Rogaeva E. A. Levesque G. Ikeda M.. (1995). Cloning of a gene bearing missense mutations in early-onset familial Alzheimer’s disease. Nature 375, 754–760. 10.1038/375754a0 7596406

[B147] Shirotani K. Edbauer D. Kostka M. Steiner H. Haass C. (2004). Immature nicastrin stabilizes APH-1 independent of PEN-2 and presenilin: identification of nicastrin mutants that selectively interact with APH-1. J. Neurochem. 89, 1520–1527. 10.1111/j.1471-4159.2004.02447.x 15189355

[B148] Siman R. Reaume A. G. Savage M. J. Trusko S. Lin Y. G. Scott R. W.. (2000). Presenilin-1 P264L knock-in mutation: differential effects on abeta production, amyloid deposition and neuronal vulnerability. J. Neurosci. 20, 8717–8726. 11102478 10.1523/JNEUROSCI.20-23-08717.2000 PMC6773081

[B149] Sisodia S. S. Kim S. H. Thinakaran G. (1999). Function and dysfunction of the presenilins. Am. J. Hum. Genet. 65, 7–12. 10.1086/302475 10364510 PMC1378068

[B150] Slowik A. Merres J. Elfgen A. Jansen S. Mohr F. Wruck C. J.. (2012). Involvement of formyl peptide receptors in receptor for advanced glycation end products (RAGE)–and amyloid beta 1–42-induced signal transduction in glial cells. Mol. Neurodegener. 7:55. 10.1186/1750-1326-7-55 23164356 PMC3519738

[B151] Sobhanifar S. Schneider B. Löhr F. Gottstein D. Ikeya T. Mlynarczyk K.. (2010). Structural investigation of the C-terminal catalytic fragment of presenilin 1. Proc. Natl. Acad. Sci. U S A 107, 9644–9649. 10.1073/pnas.1000778107 20445084 PMC2906861

[B152] Steiner H. Winkler E. Edbauer D. Prokop S. Basset G. Yamasaki A.. (2002). PEN-2 is an integral component of the gamma-secretase complex required for coordinated expression of presenilin and nicastrin. J. Biol. Chem. 277, 39062–39065. 10.1074/jbc.C200469200 12198112

[B153] St George-Hyslop P. Fraser P. E. (2012). Assembly of the presenilin gamma-/epsilon-secretase complex. J. Neurochem. 120(Suppl. 1), 84–88. 10.1111/j.1471-4159.2011.07505.x 22122073

[B154] Swiatek P. J. Lindsell C. E. del Amo F. F. Weinmaster G. Gridley T. (1994). Notch1 is essential for postimplantation development in mice. Genes Dev. 8, 707–719. 10.1101/gad.8.6.707 7926761

[B155] Tabuchi K. Chen G. Südhof T. C. Shen J. (2009). Conditional forebrain inactivation of nicastrin causes progressive memory impairment and age-related neurodegeneration. J. Neurosci. 29, 7290–7301. 10.1523/jneurosci.1320-09.2009 19494151 PMC2719251

[B156] Takagi S. Tominaga A. Sato C. Tomita T. Iwatsubo T. (2010). Participation of transmembrane domain 1 of presenilin 1 in the catalytic pore structure of the gamma-secretase. J. Neurosci. 30, 15943–15950. 10.1523/jneurosci.3318-10.2010 21106832 PMC6633739

[B157] Takami M. Nagashima Y. Sano Y. Ishihara S. Morishima-Kawashima M. Funamoto S.. (2009). gamma-Secretase: successive tripeptide and tetrapeptide release from the transmembrane domain of beta-carboxyl terminal fragment. J. Neurosci. 29, 13042–13052. 10.1523/jneurosci.2362-09.2009 19828817 PMC6665297

[B158] Tamayev R. D’Adamio L. (2012). Inhibition of gamma-secretase worsens memory deficits in a genetically congruous mouse model of Danish dementia. Mol. Neurodegener. 7:19. 10.1186/1750-1326-7-19 22537414 PMC3407505

[B160] Thinakaran G. Borchelt D. R. Lee M. K. Slunt H. H. Spitzer L. Kim G.. (1996). Endoproteolysis of presenilin 1 and accumulation of processed derivatives in vivo. Neuron 17, 181–190. 10.1016/s0896-6273(00)80291-3 8755489

[B161] Thinakaran G. Harris C. L. Ratovitski T. Davenport F. Slunt H. H. Price D. L.. (1997). Evidence that levels of presenilins (PS1 and PS2) are coordinately regulated by competition for limiting cellular factors. J. Biol. Chem. 272, 28415–28422. 10.1074/jbc.272.45.28415 9353300

[B159] Thinakaran G. Parent A. T. (2004). Identification of the role of presenilins beyond Alzheimer’s disease. Pharmacol. Res. 50, 411–418. 10.1016/j.phrs.2003.12.026 15304238

[B162] Torres M. Jimenez S. Sanchez-Varo R. Navarro V. Trujillo-Estrada L. Sanchez-Mejias E.. (2012). Defective lysosomal proteolysis and axonal transport are early pathogenic events that worsen with age leading to increased APP metabolism and synaptic Abeta in transgenic APP/PS1 hippocampus. Mol. Neurodegener. 7:59. 10.1186/1750-1326-7-59 23173743 PMC3575255

[B163] Tournoy J. Bossuyt X. Snellinx A. Regent M. Garmyn M. Serneels L.. (2004). Partial loss of presenilins causes seborrheic keratosis and autoimmune disease in mice. Hum. Mol. Genet. 13, 1321–1331. 10.1093/hmg/ddh151 15128703

[B164] Tu H. Nelson O. Bezprozvanny A. Wang Z. Lee S. F. Hao Y. H.. (2006). Presenilins form ER Ca2+ leak channels, a function disrupted by familial Alzheimer’s disease-linked mutations. Cell 126, 981–993. 10.1016/j.cell.2006.06.059 16959576 PMC3241869

[B165] van Es J. H. van Gijn M. E. Riccio O. van den Born M. Vooijs M. Begthel H.. (2005). Notch/gamma-secretase inhibition turns proliferative cells in intestinal crypts and adenomas into goblet cells. Nature 435, 959–963. 10.1038/nature03659 15959515

[B166] Veeraraghavalu K. Choi S. H. Zhang X. Sisodia S. S. (2013). Endogenous expression of FAD-linked PS1 impairs proliferation, neuronal differentiation and survival of adult hippocampal progenitors. Mol. Neurodegener. 8:41. 10.1186/1750-1326-8-41 24138759 PMC3853710

[B167] Vetrivel K. S. Gong P. Bowen J. W. Cheng H. Chen Y. Carter M.. (2007). Dual roles of the transmembrane protein p23/TMP21 in the modulation of amyloid precursor protein metabolism. Mol. Neurodegener. 2:4. 10.1186/1750-1326-2-4 17288597 PMC1803014

[B168] Vetrivel K. S. Zhang X. Meckler X. Cheng H. Lee S. Gong P.. (2008). Evidence that CD147 modulation of beta-amyloid (Abeta) levels is mediated by extracellular degradation of secreted Abeta. J. Biol. Chem. 283, 19489–19498. 10.1074/jbc.m801037200 18456655 PMC2443668

[B169] Vetrivel K. S. Zhang Y. W. Xu H. Thinakaran G. (2006). Pathological and physiological functions of presenilins. Mol. Neurodegener. 1:4. 10.1186/1750-1326-1-4 16930451 PMC1513131

[B170] von Rotz R. C. Kohli B. M. Bosset J. Meier M. Suzuki T. Nitsch R. M.. (2004). The APP intracellular domain forms nuclear multiprotein complexes and regulates the transcription of its own precursor. J. Cell. Sci. 117, 4435–4448. 10.1242/jcs.01323 15331662

[B171] Wang X. Huang T. Bu G. Xu H. (2014). Dysregulation of protein trafficking in neurodegeneration. Mol. Neurodegener. 9:31. 10.1186/1750-1326-9-31 25152012 PMC4237948

[B172] Wang H. Luo W. J. Zhang Y. W. Li Y. M. Thinakaran G. Greengard P.. (2004). Presenilins and gamma-secretase inhibitors affect intracellular trafficking and cell surface localization of the gamma-secretase complex components. J. Biol. Chem. 279, 40560–40566. 10.1074/jbc.m404345200 15247291

[B173] Wang R. Tang P. Wang P. Boissy R. E. Zheng H. (2006). Regulation of tyrosinase trafficking and processing by presenilins: partial loss of function by familial Alzheimer’s disease mutation. Proc. Natl. Acad. Sci. U S A 103, 353–358. 10.1073/pnas.0509822102 16384915 PMC1326180

[B174] Watanabe N. Tomita T. Sato C. Kitamura T. Morohashi Y. Iwatsubo T. (2005). Pen-2 is incorporated into the gamma-secretase complex through binding to transmembrane domain 4 of presenilin 1. J. Biol. Chem. 280, 41967–41975. 10.1074/jbc.m509066200 16234244

[B175] Weggen S. Eriksen J. L. Das P. Sagi S. A. Wang R. Pietrzik C. U.. (2001). A subset of NSAIDs lower amyloidogenic Abeta42 independently of cyclooxygenase activity. Nature 414, 212–216. 10.1038/35102591 11700559

[B176] Weidemann A. Eggert S. Reinhard F. B. Vogel M. Paliga K. Baier G.. (2002). A novel epsilon-cleavage within the transmembrane domain of the Alzheimer amyloid precursor protein demonstrates homology with Notch processing. Biochemistry 41, 2825–2835. 10.1021/bi015794o 11851430

[B177] Wilcock G. K. Black S. E. Hendrix S. B. Zavitz K. H. Swabb E. A. Laughlin M. A.. (2008). Efficacy and safety of tarenflurbil in mild to moderate Alzheimer’s disease: a randomised phase II trial. Lancet Neurol. 7, 483–493. 10.1016/s1474-4422(08)70090-5 18450517

[B178] Wolfe M. S. Selkoe D. J. (2014). gamma-Secretase: a horseshoe structure brings good luck. Cell 158, 247–249. 10.1016/j.cell.2014.06.043 25036627

[B179] Wolfe M. S. Xia W. Ostaszewski B. L. Diehl T. S. Kimberly W. T. Selkoe D. J. (1999). Two transmembrane aspartates in presenilin-1 required for presenilin endoproteolysis and gamma-secretase activity. Nature 398, 513–517. 10.1038/19077 10206644

[B180] Wong G. T. Manfra D. Poulet F. M. Zhang Q. Josien H. Bara T.. (2004). Chronic treatment with the gamma-secretase inhibitor LY-411,575 inhibits beta-amyloid peptide production and alters lymphopoiesis and intestinal cell differentiation. J. Biol. Chem. 279, 12876–12882. 10.1074/jbc.m311652200 14709552

[B181] Wunderlich P. Glebov K. Kemmerling N. Tien N. T. Neumann H. Walter J. (2013). Sequential proteolytic processing of the triggering receptor expressed on myeloid cells-2 (TREM2) protein by ectodomain shedding and gamma-secretase-dependent intramembranous cleavage. J. Biol. Chem. 288, 33027–33036. 10.1074/jbc.m113.517540 24078628 PMC3829152

[B182] Xia X. Qian S. Soriano S. Wu Y. Fletcher A. M. Wang X. J.. (2001). Loss of presenilin 1 is associated with enhanced beta-catenin signaling and skin tumorigenesis. Proc. Natl. Acad. Sci. U S A 98, 10863–10868. 10.1073/pnas.191284198 11517342 PMC58565

[B183] Xie T. Yan C. Zhou R. Zhao Y. Sun L. Yang G.. (2014). Crystal structure of the gamma-secretase component nicastrin. Proc. Natl. Acad. Sci. U S A 111, 13349–13354. 10.2210/pdb4r12/pdb 25197054 PMC4169925

[B184] Yang D. S. Tandon A. Chen F. Yu G. Yu H. Arawaka S.. (2002). Mature glycosylation and trafficking of nicastrin modulate its binding to presenilins. J. Biol. Chem. 277, 28135–28142. 10.1074/jbc.m110871200 12032140

[B185] Youmans K. L. Tai L. M. Kanekiyo T. Stine W. B. Jr. Michon S. C. Nwabuisi-Heath E.. (2012). Intraneuronal Aβ detection in 5xFAD mice by a new Aβ-specific antibody. Mol. Neurodegener. 7:8. 10.1186/1750-1326-7-8 22423893 PMC3355009

[B186] Yu G. Chen F. Levesque G. Nishimura M. Zhang D. M. Levesque L.. (1998). The presenilin 1 protein is a component of a high molecular weight intracellular complex that contains beta-catenin. J. Biol. Chem. 273, 16470–16475. 10.1074/jbc.273.26.16470 9632714

[B187] Yu G. Nishimura M. Arawaka S. Levitan D. Zhang L. Tandon A.. (2000). Nicastrin modulates presenilin-mediated notch/glp-1 signal transduction and betaAPP processing. Nature 407, 48–54. 10.1038/35024009 10993067

[B188] Zhang Y. W. Luo W. J. Wang H. Lin P. Vetrivel K. S. Liao F.. (2005). Nicastrin is critical for stability and trafficking but not association of other presenilin/gamma-secretase components. J. Biol. Chem. 280, 17020–17026. 10.1074/jbc.m409467200 15711015 PMC1201533

[B189] Zhang H. Sun S. Herreman A. De Strooper B. Bezprozvanny I. (2010). Role of presenilins in neuronal calcium homeostasis. J. Neurosci. 30, 8566–8580. 10.1523/JNEUROSCI.1554-10.2010 20573903 PMC2906098

[B190] Zhang Y. W. Thompson R. Zhang H. Xu H. (2011). APP processing in Alzheimer’s disease. Mol. Brain. 4:3. 10.1186/1756-6606-4-3 21214928 PMC3022812

[B191] Zhang Y. W. Wang R. Liu Q. Zhang H. Liao F. F. Xu H. (2007). Presenilin/gamma-secretase-dependent processing of beta-amyloid precursor protein regulates EGF receptor expression. Proc. Natl. Acad. Sci. U S A 104, 10613–10618. 10.1073/pnas.0703903104 17556541 PMC1888796

[B192] Zhang C. Wu B. Beglopoulos V. Wines-Samuelson M. Zhang D. Dragatsis I.. (2009). Presenilins are essential for regulating neurotransmitter release. Nature 460, 632–636. 10.1038/nature08177 19641596 PMC2744588

[B193] Zhao G. Mao G. Tan J. Dong Y. Cui M. Z. Kim S. H.. (2004). Identification of a new presenilin-dependent zeta-cleavage site within the transmembrane domain of amyloid precursor protein. J. Biol. Chem. 279, 50647–50650. 10.1074/jbc.c400473200 15485850

[B194] Zhao G. Tan J. Mao G. Cui M. Z. Xu X. (2007). The same gamma-secretase accounts for the multiple intramembrane cleavages of APP. J. Neurochem. 100, 1234–1246. 10.1111/j.1471-4159.2006.04302.x 17241131

[B195] Zhou S. Zhou H. Walian P. J. Jap B. K. (2005). CD147 is a regulatory subunit of the gamma-secretase complex in Alzheimer’s disease amyloid beta-peptide production. Proc. Natl. Acad. Sci. U S A 102, 7499–7504. 10.1073/pnas.0502768102 15890777 PMC1103709

